# Lipid Emulsion to Treat Acute Poisonings: Mechanisms of Action, Indications, and Controversies

**DOI:** 10.3390/pharmaceutics15051396

**Published:** 2023-05-03

**Authors:** Karim Jaffal, Lucie Chevillard, Bruno Mégarbane

**Affiliations:** 1 Department of Medical and Toxicological Critical Care, Federation of Toxicology, Lariboisière Hospital, 75010 Paris, France; 2 INSERM UMRS-1144, Paris-Cité University, 75006 Paris, France

**Keywords:** lipid emulsion, Intralipid^®^, poisoning, cardiotoxicant, detoxification, cardiovascular failure, shock, cardiac arrest, lipid sink

## Abstract

Biodetoxification using intravenous lipid emulsion (ILE) in acute poisoning is of growing interest. As well as for local anesthetics, ILE is currently used to reverse toxicity caused by a broad-spectrum of lipophilic drugs. Both pharmacokinetic and pharmacodynamic mechanisms have been postulated to explain its possible benefits, mainly combining a scavenging effect called “lipid sink” and cardiotonic activity. Additional mechanisms based on ILE-attributed vasoactive and cytoprotective properties are still under investigation. Here, we present a narrative review on lipid resuscitation, focusing on the recent literature with advances in understanding ILE-attributed mechanisms of action and evaluating the evidence supporting ILE administration that enabled the international recommendations. Many practical aspects are still controversial, including the optimal dose, the optimal administration timing, and the optimal duration of infusion for clinical efficacy, as well as the threshold dose for adverse effects. Present evidence supports the use of ILE as first-line therapy to reverse local anesthetic-related systemic toxicity and as adjunct therapy in lipophilic non-local anesthetic drug overdoses refractory to well-established antidotes and supportive care. However, the level of evidence is low to very low, as for most other commonly used antidotes. Our review presents the internationally accepted recommendations according to the clinical poisoning scenario and provides the precautions of use to optimize the expected efficacy of ILE and limit the inconveniences of its futile administration. Based on their absorptive properties, the next generation of scavenging agents is additionally presented. Although emerging research shows great potential, several challenges need to be overcome before parenteral detoxifying agents could be considered as an established treatment for severe poisonings.

## 1. Introduction

Biodetoxification using drug scavenging agents is of growing interest, as drug overdoses represent a major health problem accounting for thousands of deaths annually worldwide, most often among the young. Poisoning is responsible for more than 3 million calls to US poison control centers annually [1]. Two thirds of these calls are in relation to the ingestion of overdosed prescription drugs, with major fatality risk if cardiovascular medications are involved.

The use of intravenous lipid emulsion (ILE) as an antidote to reverse local anesthetic-related systemic toxicity (LAST) has gained widespread support following convincing data from successful case reports and animal studies [2]. ILE was suggested as a promising agent for poisonings involving lipophilic agents, especially if unresponsive to the recommended therapies [3]. Its easy administration in emergent conditions at the bedside and its original properties, supporting its ability to alter both the pharmacokinetics (PK) and the pharmacodynamics (PD) of lipophilic toxicants synergistically with supportive care, antidotes, and decontamination and elimination enhancement techniques, opened up new perspectives for emergency physicians and intensivists to improve the management of severe poisonings. To date, ILE has been used in poisoned patients to reverse cardiac toxicity induced by a broad spectrum of drugs (Table 1) [4,5].

Evidence for ILE use in poisoning is evolving. Several mechanisms of action have been hypothesized [6], including i: intravascular sequestration of the toxicant and its enhanced redistribution to biologically inert tissues; ii: augmentation of fatty acid utilization for adenosine triphosphate (ATP) synthesis; and iii: direct cardiotonic and ion channel effects. However, much remains to be elucidated. Uncertainties are still present regarding the optimal composition, dosing, mechanisms of action, and efficiency of ILE. Without clarification of the ILE mechanism of action, caution should remain regarding its role and indications. We present a mini review of the recent literature, focusing on the advances in ILE mechanism understanding and evaluating the evidence supporting ILE administration in clinical toxicology. This review aims to clarify ILE indications as rescue therapy in the severely poisoned patient to help the clinician at the bedside in anticipating its expected efficacy and adverse effects and improving patient management.

## 2. Rationale for ILE Use in Clinical Toxicology

With functional toxicants, the most common approach to manage poisoning is to provide the support needed to maintain organ function, relying on the endogenous ability to metabolize and eliminate the toxicant. Gastrointestinal decontamination, including gastric lavage and activated charcoal administration, can minimize absorption, but these techniques are controversial and poorly effective more than 1–2 h post-ingestion [7,8]. Extracorporeal techniques such as hemodialysis and hemofiltration to enhance drug elimination are of questionable clinical pertinence for drugs with large volumes of distribution and/or high protein binding affinities. Finally, antidotes are necessary in combination with supportive care when the drug is associated with a high mortality rate or poor long-term outcome [9].

Numerous experimental and clinical studies have established that ILE can increase the blood tissue partitioning of lipophilic drugs, improve cardiac performance, and result in relevant beneficial effects such as post-conditioning, direct inotropy, and the activation of cytoprotective pathways [3,5,6]. By contrast, randomized controlled trials (RCTs), considered as the best evidence to support the benefits of ILE, are rare due to obvious ethical difficulties and the rarity of clinical indications. Therefore, to date, mainly experimental and case report data provide support for the possible efficacy of ILE in clinical toxicology in selected indications. [5]. While awaiting human data, one approach to obtain approval for therapy in acute poisoning is the « animal efficacy and human safety » rule.

### 2.1. A Success Story

In 1962, after the commercial release of Intralipid^®^ (Fresenius Kabi, Bad Homburg, Germany), clinicians proposed the use of intravenous (IV) fat emulsions as drug binders or components of extracorporeal lipid dialysis. Russell et al. reported that IV cottonseed oil could shorten the duration of thiopental anesthesia [10]. Adding olive or cottonseed oil to the dialysate was shown to enhance glutethimide removal [11], and one camphor-intoxicated patient was treated successfully with lipid extracorporeal hemodialysis [12]. In the following years, devices for lipid dialysis were developed, and proposals of more specifically designed dialysates for detoxification were postulated [13,14]. Laboratory investigations demonstrated that corn oil addition to dialysates was effective at moving drugs (e.g., imipramine, amitriptyline, and glutethimide) out of the plasma and into the dialysate [15]. Krieglstein et al. were the first to demonstrate that a marketed fat emulsion (10% Lipofundin^®^, B. Braun, Taguig City, Philippines) could bind chlorpromazine in vitro and save rats from lethal chlorpromazine toxicity [16]. Thereafter, investigations focused on the underlying mechanistic actions of ILE.

Weinberg et al. published their original findings demonstrating that resuscitation or pretreatment with ILE resulted in the improvement of cardiac toxicity associated with bupivacaine in rats and dogs [17,18]. Following these supportive animal works, ILE was administered in human cases of cardiac arrest and neurologic toxicity attributable to LAST, with successful resuscitation after the failure of conventional resuscitation [2]. Nearly two decades later, the majority of academic societies recommend ILE as a first-line treatment of LAST [19].

### 2.2. ILE Constituents

**Figure 1:**
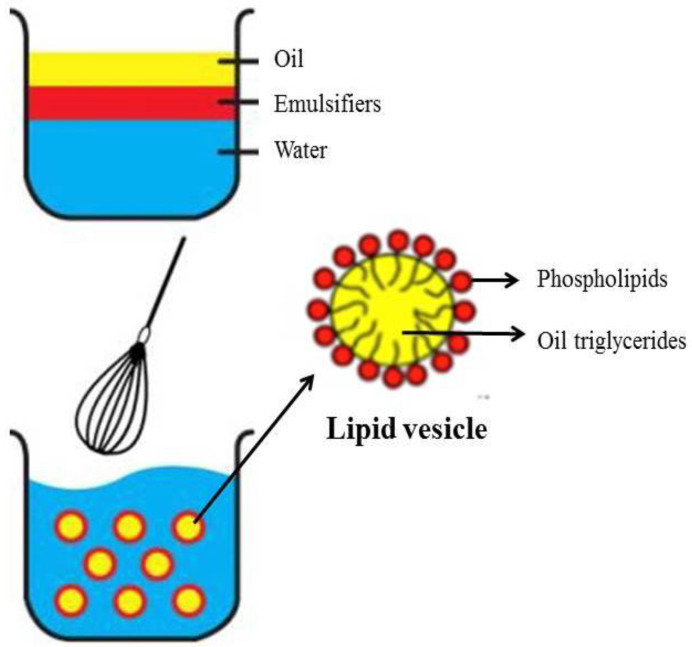
The making of a lipid emulsion. Droplets have a monolayer of phospholipids as emulsifier, which separates the interior lipid phase from the exterior aqueous phase.

ILEs are sterile nanometer-sized droplets of triglyceride oils in water stabilized by phospholipid surfactants [20]. The average particle size is between 200 and 600 nm, depending on the method and the emulsion. Phospholipids such as egg lecithin are added as emulsifiers to improve fat solubility. Emulsifiers, which are both fat- and water-soluble, surround the lipid droplet (Figure 1). Sodium hydroxide is added to adjust the pH. Currently marketed products have a pH between 6.0 and 9.0 and a ~270 mOsm/L osmolality, and are ready to infuse using peripheral vein access.

ILEs may contain varying types (soybean, coconut, olive, and fish) and concentrations of triglyceride oils (10, 20, and 30%). Droplets have similar shapes and sizes to physiological chylomicrons and are similarly metabolized. Fatty acids represent the lipid phase of ILE with neutral long-chain triglycerides (LCTs) or a mix of medium-chain triglycerides (MCTs) and LCTs.

Intralipid^®^ is the most often studied product among ILEs. It is an emulsion containing 20% soybean oil, 2.25% glycerin, and 1.2% egg phospholipids [21]. Intralipid^®^ is currently manufactured with lipid concentrations of 10, 20, and 30% and commonly used for parenteral nutrition, drug carrier vehicles, lipid rescue therapy, and ischemia-reperfusion injury attenuation therapy [21]. Although Intralipid^®^ is the most often administered lipid rescue therapy, the use of other products has been reported [22,23]. The composition is of importance since it influences the scavenging properties and the side effects of ILE.

### 2.3. The Absorptive Properties

ILEs are useful carriers for lipophilic drugs [23]. They are used to deliver drugs such as propofol, etomidate, diazepam, and amphotericin B [24] and more recently as highly efficient and targeted delivery systems for cancer therapy [25]. Based on their absorptive properties, lipophilic drugs can diffuse across the phospholipid coating into the blood when the purpose is to deliver the drug or out of the blood when ILE is used to treat drug overdose [26,27]. The principles of Fick’s law govern drug diffusion according to the concentration gradient across the lipid droplet membrane, the surface area of the drug-containing lipid droplet, and the drug partition coefficient between the oil and aqueous phases. The amount of toxicant that can be sequestered by a given therapy is measured by its partition coefficient, which is the concentration ratio of the toxicant dissolved in the oil over the aqueous phase at equilibrium. The toxicant can also be adsorbed on the emulsion surface. Regarding the adsorption of the toxicant, the interfacial dynamics of droplet–poison binding are unknown, and phospholipid micelles likely exist in aqueous solution alongside the emulsified oil droplets and could interact with the toxicant. Of note, neither of these phenomena are understood adequately and both deserve further study. Each drug and metabolite has different degrees of plasma protein binding and partition coefficients depending on the physicochemical conditions. For example, a drop in arterial pH to 7.0 tends to reduce bupivacaine protein binding, and the resulting increase in free bupivacaine may allow lipid scavengers to play a more significant role in the drug uptake [28,29].

**Figure 2:**
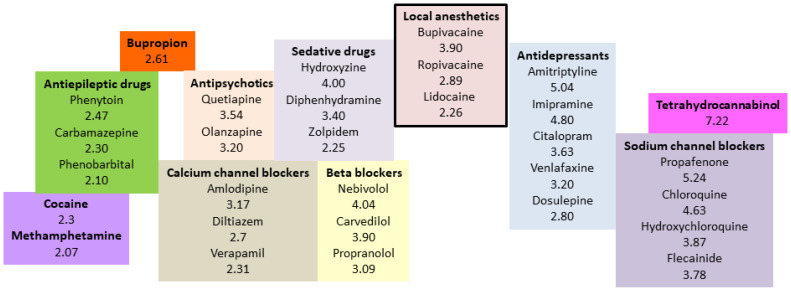
Partition coefficients of some selected lipophilic drugs for which the use of lipid emulsion could be considered in poisoning.

Of note, the octanol/water partition coefficient (LogP) used to reflect lipophilicity (Figure 2) differs from the actual drug partitioning between the serum and lipid emulsion. LogP applies only to the neutral moiety, which, at equilibrium, might be present in a very low concentration compared with the charged version. At physiological pH, several drug candidates for ILE-based detoxification are predominantly charged. The lipid solubility of charged compounds is negligible; but these drugs could potentially partition on the surface through electrostatic interactions with charged phospholipid surfactants in the emulsion. Intralipid^®^ 20% was reported to have a zeta potential between −45 and −40 mV, indicating that the emulsion has significant surface charge [30]. Recently, the distribution coefficient (LogD) was suggested to be a better predictor of the actual equilibrium behavior of drugs [31]. LogD accounts for both the non-ionized and ionized forms of a drug in both the octanol and water phases. This ratio is more difficult to measure and depends on the pH of the system evaluated. It may also be a better predictor of drug lipophilicity and sequestration.

Recently, two other new parameters, i.e., drug accommodation capacity and drug capture kinetics, were described to characterize the drug capture capability of lipid emulsions and their resulting effects on PK in overdose [32]. Drug capture prediction based on these two parameters was shown to be more accurate than LogP when considering the lipid emulsion-attributed reduction in the half-life and area under the drug concentration-time curve.

To summarize, the charged portion of ionized drugs could interact with the surface of lipid droplets through electrostatics, while the uncharged portion could interact with the oily interior and the phospholipid bilayers to maintain drug sequestration. The clinical utility of these absorptive properties (hydrophobic interior, phospholipid membrane, and surface charges) lies in increasing drug sequestration to provide recovery from toxicity. Various predictors of drug capture have been proposed, including LogP, LogD, and drug accommodation capacity. However, the best predictor, if considering their emergent availability at the bedside versus their prediction accuracy, remains debated.

## 3. Mechanisms of Action

**Figure 3:**
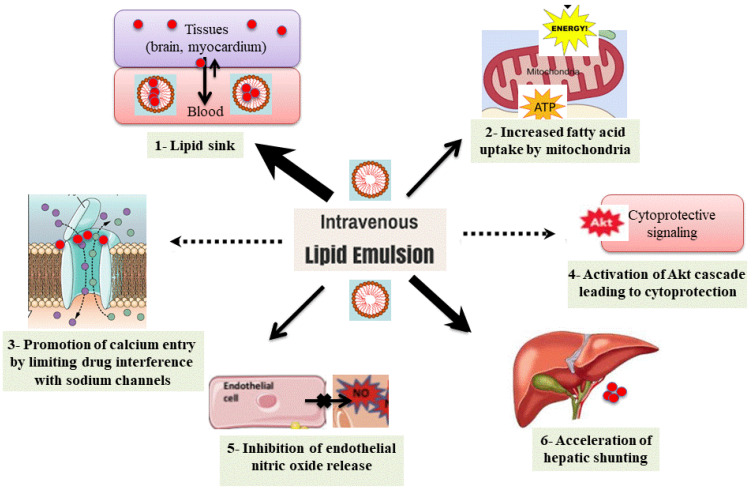
Suggested mechanisms for lipid resuscitation (adapted from Weinberg GL [6]).

The mechanisms responsible for ILE-based detoxification remain the subject of ongoing extensive studies and debate [33,34,35]. Both PK and PD mechanisms have been postulated to explain the possible antidotal action of ILE; however, the relative contribution of each mechanism remains uncertain. A multimodal synergy between the proposed mechanisms may exist. Depending on the drug, patient’s conditions, and delay in initiation, these mechanisms may variably contribute to the clinically observed improvement. Six postulated mechanisms have been hypothesized (Figure 3) [12].

### 3.1. The Lipid Sink Theory

The “lipid sink” or “PK sequestration” theory is the one most studied. Triglyceride oils in ILE exhibit a high affinity for lipophilic drugs and create a lipid-rich PK compartment within the bloodstream, drawing them away from their targets and free blood. A new equilibrium is established thereafter, reducing tissue concentrations and helping to restore organ function, allowing the drug’s metabolism and elimination. ILE inactivates a portion of lipophilic drugs by acting as alternative binding surfaces, making them unavailable to act on their target organs [17].

ILE has been reported to reverse the toxicity caused by drugs lacking any common mechanism, site of action, chemical structure, or clinical effects except lipophilicity [6]. The partitioning effect is in accordance with lipid dialysis therapy [11], and has been demonstrated in numerous in vitro studies [36,37,38,39,40]. French et al. measured the amount of several drugs sequestered in vitro from human serum using Intralipid^®^ [41]. The only significant factors were the reported LogP and the volume of distribution of each drug. Consistent, ex vivo studies also showed that radiolabeled bupivacaine levels declined more rapidly in lipid-infused rat hearts compared with lipid-free control groups [42,43].

The infused lipid emulsion appears in the blood as emulsified oil droplets acting on the lipophilic drug-induced cardiovascular failure through (1) redistribution of the toxicant from its targeted tissues (lipid sink theory, the main acknowledged mechanism to date), (2) mitochondrial increase in fatty acid uptake and enhanced cell energetic production (metabolic theory) (3) limitation of the toxicant interference with the sodium channels, thus promoting calcium entry via voltage-dependent calcium channels (membrane and ionotropic theory), (4) activation of protein kinase B (called Akt), leading to the cytoprotective signaling cascade (cytoprotection theory), (5) attenuation of endothelium-dependent nitric oxide-mediated vasodilation and post-ischemic vasodilation by decreasing nitric oxide bioavailability (nitric oxide theory), and (6) acceleration of liver shunting (pharmacokinetic theory). The arrow thickness represents the suggested probability rate of each postulated mechanism based on the literature.

In vivo studies supported a substantial contribution of partitioning in successful lipid resuscitation. Niiya et al. found that pretreating pigs with lipids protected them against amiodarone-induced hypotension [44]. The lipid-free aqueous phase exhibited lower amiodarone concentrations in ILE-treated pigs than controls receiving saline. Furthermore, ultra-centrifuging the plasma to allow separation of the lipid-bound drug indicated that amiodarone was preferentially partitioned into the newly formed lipid phase. Weinberg et al. analyzed rat blood samples, comparing vasopressin with Intralipid^®^ resuscitation of LAST [43]. Bupivacaine was present in higher concentrations in the aqueous phase (lipid-free) of the vasopressin- than the ILE-treated rats. Lipid treatment resulted in lower aqueous and myocardial bupivacaine concentration, leading to a better cardiac performance. Litonius et al. demonstrated increased total amitriptyline concentrations with a decrease in free amitriptyline fraction when ILE was given after amitriptyline infusion in amitriptyline-poisoned pigs [45]. In a rabbit model of IV clomipramine toxicity, increased total blood clomipramine concentrations concomitantly occurred with improved blood pressures following ILE infusion, consistent with the toxicant sequestration into the intravascular lipid phase [46]. Indirect evidence included the fact that lipids could reverse both neurologic and cardiac toxicity, though the brain does not metabolize fatty acids as an energy source [6]. Finally, PK data provided some evidence to support this theory [18,46,47,48], together with human case reports [49,50]. A shift in partitioning coefficients for the heart and brain was observed in ILE-treated bupivacaine-poisoned animals, which recovered independently from any effect on bupivacaine metabolism, supporting the ILE-dependent partitioning theory [19].

Emerging evidence suggests that the application of the sink theory should be approached differently. With improved understanding of lipid resuscitation, ILE droplets are viewed as a lipid shuttle responsible for a capture/release mechanism to move a drug around and not only as a sink that captures and isolates the drug. A newly formed IV lipid compartment transiently sequesters the drug and accelerates its movement from drug-susceptible organs such as the brain and heart to organs that can store (muscle, adipose), metabolize (liver), and excrete (kidney, bladder) the drug [19,51]. A transient increase followed by subsequent decrease in blood concentrations of lipophilic drugs after ILE infusion has been reported in animals [51] and humans [52,53,54,55]. Interestingly, Shi et al. reported decreased bupivacaine concentrations in the target organs of intoxicated rats in addition to increased liver bupivacaine concentrations following ILE infusion [51]. The measured elimination half-life decreased and the clearance increased, consistent with the hypothesis of augmented redistribution. Fettiplace et al. demonstrated the occurrence of PK sequestration combined with cardiotonic effects in rats dosed with toxic bupivacaine concentrations before ILE administration [19]. They reported a rapid detoxifying effect related to ILE, acting primarily on targeted organs (heart and brain) dependent on the ILE partitioning effect. Once the drug concentration in the heart fell below a toxic threshold, ILE produced cardiotonic effects based on the combined actions of volume expansion and direct inotropic properties. Subsequently, improved cardiac output combined with increased blood carriage was shown to enhance drug redistribution to sites of metabolism and storage. PD parameters were measured, and several PK/PD models for bupivacaine (accounting for various mechanisms of ILE action) were considered. The authors reported that the best model agreement was achieved when both the sequestering and cardiotonic effect were included. They also extended their theory to account for an accelerated metabolic effect with ILE therapy, but metabolism was not required for recovery [19]. These studies clearly demonstrated that ILE provides multiple but not necessarily independent mechanisms of action to allow recovery from overdose.

Nevertheless, caution is needed, as PK sequestration does not always lead to reduced toxicity. Many questions remain unresolved. The lipid-rich compartment provided by ILE can actually increase the gastrointestinal absorption of lipophilic drugs, which may strengthen the toxicity, as shown in a model where ILE was administered before cardiovascular toxicity onset in contrast to the typical ILE use in humans [56,57]. ILE may facilitate the gastrointestinal absorption of lipophilic drugs administered orally in overdose such as amitriptyline or verapamil and thereby aggravate the poisoning. However, the exact molecular mechanism by which ILE enhances drug absorption remains unknown. It could be related to the increased drug concentration gradient between the gastrointestinal interstitial fluid and the systemic blood containing ILE, which exhibits high affinity to the drug. Another hypothesis was also postulated based on these experimental observations [56,57]: ILE may retardate drug redistribution to low-blood flow organs, resulting in a higher exposure of high-blood flow organs such as the brain and the heart for a longer period and in subsequent increased drug toxicity. The ILE treatment of oral versus parenteral overdoses likely differs, especially when considering the timing and rate of infusion after the initial bolus. Since ingestion remains the main route of overdose in humans, studies are urgently required to understand how ILE can modify the PK in oral overdose. It is noteworthy that in some situations, toxicity may be related not to the toxicant itself but to its metabolites. The detoxification and excretion pathways may become saturated, making the lipid sink ineffective. Finally, the half-life of lipid droplets, the possible release from the adipose tissue, and the interaction with the concomitant medications need to be investigated [58].

PK models assert that a scavenging effect alone cannot account for the rapid recovery from toxicity occurring after ILE administration [48]. Continued cardiopulmonary resuscitation efforts maintaining efficient circulation are important to allow accelerated redistribution until the effects of ILE occur. Drugs for which ILE detoxification have been used have different chemical properties and PK profiles. A dominant mechanism for all cases is unlikely. Certainly, PK evidence alone is insufficient to validate ILE as a proposed detoxification therapy.

### 3.2. The Cardiotonic Effects

Only the scavenging effect and a direct cardiac effect considered together can explain the rapid recovery from drug toxicity [19,59]. ILE is able to exert direct physiological effects on the heart and vasculature, improving cardiac output [59,60], which may facilitate the accelerated redistribution of the toxicant.

The underlying mechanisms allowing ILE-attributed improvement in cardiac output have not been fully elucidated. The infused volume of ILE is a major issue but not the only one, and other contributors are still unclear. The calcium influx and fatty acid hypothesis is the most popular. Fatty acids are supposed to increase calcium influx in the myocardial cells to produce a positive inotropic effect [61]. The application of both saturated and unsaturated long-chain free fatty acids has been shown to activate voltage-gated calcium channels in myocardial isolates [34].

A large lipid load could offset the potent inhibition of fatty acid metabolism caused by the toxicant, providing a source of energy to the myocardial cells [62]. Studies demonstrated that bupivacaine disrupts the targets of classical insulin signaling, including protein kinase B (Akt), and activates other controllers of glucose homeostasis, including 5′-adenosine monophosphate activated protein kinase (AMPK). Both kinases integrate signaling modulating sensitivity to endogenous insulin, leading to glycogen accumulation in the cardiac tissue. Signaling sensitization serves as a protective mechanism to normalize energy processing in settings where metabolism is impaired, leading to myocardial contractility improvement during recovery [63]. This hypothesis mainly depends on the timing of ILE administration with regard to the ischemia/reperfusion event [64]. This « energetic–metabolic effect » could explain how ILE may provide benefits in poisonings with less lipophilic drugs [19]. Although attractive, evidence to support this possible mechanism of ILE effects is still poor, requiring additional investigations at the cellular and molecular levels.

### 3.3. Other Mechanisms

The role of the vasoconstrictive properties of ILE on the vasculature has been established. However, the relative cardiac versus vascular contribution to the final effects is unclear [65]. ILE can attenuate the endothelium-dependent nitric oxide-mediated vasodilation and post-ischemic vasodilation by decreasing nitric oxide bioavailability [66,67,68]. Positive adrenergic sensitization is one possible hypothesis to explain ILE-induced vasoconstriction [69]. In addition, fatty acids have direct effects on sodium and calcium channels, which specifically inhibit the antagonism of local anesthetics on sodium channels or limit the action of calcium channel blockers [70,71,72]. Sodium channel antagonists are the most prominent toxicants involved in the cases with positive outcomes attributed to ILE, with reported rapid improvements in QRS duration [4,73,74].

ILE infusion attenuates cardiac ischemia reperfusion injury. Post-ischemic infusion of lipids in rodents, as observed in the experiments with metabolic inhibitors, reduced the likelihood of mitochondrial permeability transition and apoptosis [75,76]. Ischemic reperfusion injury was limited by the inhibition of mitochondrial permeability transition pores, resulting from glycogen synthase kinase-3β phosphorylation by various kinases (Akt, phosphoinositide-3 kinase, and extracellular signal-regulated ones). The activation of these cytoprotective pathways likely contributes to the clinically observed benefit of ILE in poisoning [77,78,79].

### 3.4. Contribution and Limits of Experimental Models

A meta-analysis of animal studies (*n* = 16 studies) supported the benefits of ILE use in combination with life-support measures to treat LAST, especially in relation to bupivacaine [80]. This study showed a significant ILE-attributed reduction in mortality (odds ratio, 0.24; 95% confidence interval, 0.1–0.56, *p* = 0.0012) and features of drug toxicity. Similarly, in a model of amlodipine-poisoned rats, the survival rate and hemodynamics were improved with ILE in comparison to controls and methylene blue, although the beneficial effects were small [81]. In a guinea pig model of amitriptyline toxicity, ILE improved hemodynamics, supported by fast sodium current suppression in ventricular cardiomyocytes of isolated perfused hearts [82]. Interestingly, a systematic review of available animal studies (*n* = 58 studies) underlined the study heterogeneity and limitations, mainly in relation to the experimental resuscitation conditions such as the appropriate airway management and chest compressions in models including cardiac arrest [83]. Swine did not derive the same benefits as non-swine in LAST models, due to the infused ILE dose, to the experimental design, and to the onset of anaphylactoid reactions to lipids. Non-swine models presented a more homogeneous benefit with all toxins. Therefore, researchers are still calling for additional experimental data to clarify the methodological issues in swine and confirm the role of ILE in non-anesthetic drug overdose.

## 4. Current Recommendations and Practical Considerations

### 4.1. Recommendations

Beyond the accepted setting of LAST, ILE is still under investigation as a detoxification therapy for lipophilic non-local anesthetics, including tricyclic antidepressants, beta-blockers, calcium channel blockers, and cocaine. However, even though multiple clinical reports using ILE therapy with successful detoxification are encouraging, there is no universally positive consensus due to the undetermined mechanisms of action for detoxification, concerns over the routes of intoxication, lack of human trials, the underreporting of unsuccessful treatments, and all kinds of bias in the published literature.

The Lipid Emulsion Workgroup experts (representing the American Academy of Clinical Toxicology, the European Association of Poison Centres and Clinical Toxicologists, the Asia Pacific Association of Medical Toxicology, the American College of Medical Toxicology, the American Association of Poison Control Centers, and the Canadian Association of Poison Control Centers) recently assessed the evidence regarding ILE use in acute poisoning [84]. The rigorous methodology of the Grading of Recommendations Assessment, Development, and Evaluation (GRADE) approach was used [85,86]. Two sub-groups prepared in-depth reviews regarding ILE for LAST [2] and toxicity attributed to non-local anesthetics [5].

Despite the multiple reports encouraging the use of ILE in the management of acute poisonings involving cardiotoxicants, the voting panel found an absence of evidence to recommend its use in most poisonings and clinical scenarios where its use was previously reported. Thus, the preponderance of neutral votes (resulting from a balance between pro and con assessments rather than a lack of data, which would result in no recommendation at all) likely represents the workgroup’s caution in making recommendations for or against a therapy where so few moderate- or high-quality human data existed [84]. The studies published are very heterogeneous. Due to the limitations regarding the two available RCTs [87,88], the variability in results among the studies related to the amount of ILE administrated (bolus and infusion dose, time of administration), the absence of exact measured changes, and the interval between ingestion and hospital admission, no definitive conclusions with meaningful interpretation were reached.

The working group carefully considered several specific questions for 22 toxins. When should ILE be the first-line therapy? Is ILE the drug of choice in cardiac arrest or life-threatening toxicity? Should we ever use ILE in non-life-threatening toxicity of specific drugs? For any detailed analysis of the studies and case reports, the experts directed readers to several recent reviews cited above [2,5,84]. Table 2 summarizes these recommendations:

- In bupivacaine-induced cardiac arrest, ILE after standard ACLS started (1D) was recommended (strong agreement), while the recommendation was neutral (equipoise between risk and benefit) regarding its use in cardiac arrest due to other local anesthetics;
- In case of cardiac arrest related to other local anesthetics, no recommendation was provided because of a lack of evidence. The considerable variability in lipophilicity and toxicity profiles of the other local anesthetics invalidated recommendations made by analogy rather than data. However, in the risk/benefit balance, harm appeared to be very low, leading to a neutral vote in this setting, meaning that ILE could be used in first line therapy;
- In life-threatening toxicity due to bupivacaine, the experts suggested using ILE as part of treatment modalities (2D) and recommended its use for all other local anesthetics if other therapies fail or as a last resort (1D);
- In non-life-threatening toxicity due to bupivacaine or other local anesthetics, the recommendation regarding ILE use was neutral;
- In cardiac arrest in relation to all other drugs (Class 1 Vaughan–Williams antiarrhythmic drugs, baclofen, olanzapine, other antipsychotics, selective serotonin reuptake inhibitors, ivermectin, malathion, and other pesticides/insecticides), the recommendation regarding ILE use was neutral;
- In life-threatening toxicity due to Class 1 Vaughan–Williams antiarrhythmic drugs, baclofen, ivermectin, and selective serotonin reuptake inhibitors, the recommendation regarding ILE use was neutral;
- In life-threatening toxicity due to malathion and other pesticides/insecticides, olanzapine, and other antipsychotics, the experts suggested not using ILE as a first-line therapy (2D);
- In non-life-threatening toxicity due to all these drugs, malathion, and other pesticides/insecticides, the experts suggested not using ILE as a first-line therapy (2D).

### 4.2. Practical Considerations

Many practical aspects of ILE therapy have not been validated in the clinical setting: the optimal dose for clinical efficacy, the threshold dose for adverse effects, and the minimum or maximum duration of infusion [3,5,6]. There is a lack of evidence to support any particular approach. In the case of rapid endogenous clearance, no studies evaluated the benefit of an infusion after a bolus versus a bolus alone. The most recommended dose regimen was an initial IV bolus of 1.5 mL/kg ILE followed by an infusion of 15 mL/kg/h for 30 min, with the possibility for additional boluses given at 5 min intervals if required, but this protocol was arbitrary [5,84]. However, the dose regimen could obviously depend on the toxicant and the route of intoxication. Therefore, understanding the PK, the PD, and possible PK alterations resulting from different infusion paradigms for a given drug is important to optimize and/or simplify the recommended regimen, which should be determined in basic models and validated thereafter in RCTs to obtain the highest level of evidence. Regarding ILE formulations, the voting panel agreed that there were insufficient data to discuss formulations other than 20% Intralipid^®^ in human poisonings until comparative studies could be reported [84]. Of note, rare studies reported the effectiveness of other ILEs [89], while the majority of studies recommended the use of Intralipid^®^ [36].

Data on ILE dose/response relationships to reverse toxicity in humans are insufficient to determine the optimal duration of ILE therapy,. There is a varied range of bolus doses, infusion rates, and durations reported in the literature, precluding any accurate analysis of the optimal dose regimen. Due to the lack of historical data indicating a recurrence of toxicity once clinical improvement occurs, termination of the infusion should be considered when sustained clinical improvement is obtained or, if this endpoint takes time, until the maximum dose has been reached. However, this is speculative, and studies are needed [90]. To avoid lipid overload, it was suggested that ILE doses should not exceed 10% of total blood volume to limit complications arising from an increase in triglyceride concentration (excess of 15 mmol/L) [91,92]. Additionally, fluid overload should be avoided, as it represents an increasing concern in resuscitated patients receiving ILE [93]. The appropriate time to administer ILE is also unknown. Drug–drug interactions between ILE, concomitant therapies, and the ingested toxicant represent a potential issue [56,94]. Finally, there is no consensus on whether it is useful to administer ILE as early as the prodromal signs of acute intoxication occur, including in the case of LAST [95].

ILE stability and sterilization do not represent actual issues. In the poisoned patient, ILE is infused from the manufactured package using a central or peripheral venous catheter with no particular manipulation by caregivers, contrasting with the conditions of its administration for parenteral nutrition. Preserving a stable emulsion with ~300 nm droplet size and a relatively narrow globule size distribution (200–600 nm) is important. No study specifically investigated the emulsion stability requirement in the conditions of ILE infusion to poisoned patients.

### 4.3. Safety Issues

Complications following ILE administered as part of long-term parenteral nutritional are well known (Table 3).

Those related to ILE administration as « lipid rescue » have been recently reviewed [96]. Such adverse events are important to consider when clinicians need to make a risk/benefit analysis, which seems to be proportional to the infusion rate and to the total dose received. Reporting all adverse events associated with ILE administration in the current clinical toxicology literature is needed, as significant adverse events may occur rarely and require thousands of uses. Clinicians should be aware of the expected onset of laboratory abnormalities following ILE infusion. The measurement of a number of common analytes can be markedly affected by lipemia resulting from ILE. A lack of appreciation of this effect may lead to unintentional treatment errors [92]. It is, therefore, essential to sample blood before lipid perfusion, perform PK studies, and communicate with the laboratory. They might be able to spin plasma once ILE is administered before analyzing and interpreting the results in cases of very lipemic specimens. A recent laboratory RCT showed that nearly all tests performed on a Cobas 8000-platform (Roche Diagnostics, Meylan, France) of samples obtained from ILE-treated healthy participants failed [97]. However, the ILE effects were limited to the 60 min time point, and consequences were found to be marginal, particularly regarding alkaline phosphatase, bilirubin, phosphate, and carbamide. All results could be retrieved manually.

Finally, the use of extracorporeal membrane oxygenation (ECMO) after or during lipid rescue may be associated with fat deposition in the circuits and increased blood clot formation [98]. ILE may render renal replacement therapy difficult because of filter collapse or complete occlusion from severe lipemia [99,100]. In a case report, ECMO flow rapidly deteriorated due to the lipid deposition on the circuit oxygenator despite appropriate systemic anticoagulation, volume status, and cannula position, as well as team efforts to aspirate blood from the oxygenator monitoring ports [100].

### 4.4. Clinical Uncertainties

Numerous meta-analyses, narrative reviews, and national guidelines exist, with slightly different and sometimes contradictory conclusions compared to those of the Lipid Emulsion Workgroup experts [101,102,103,104,105]. Interestingly, a quantitative meta-analysis of the same data set came to a different conclusion regarding evidence in support of ILE efficacy for LAST [80]. A meta-analysis including six RCTs conducted in acute organophosphate pesticide poisoning suggested that ILE-based resuscitation likely improved prognosis and patients’ liver function [106]. By contrast, a more recent Indian RCT showed no benefit in this setting when investigating ILE-induced changes in total atropine dose requirement over the first 24 h to treat the cholinergic crisis in addition to standard care [107]. Other RCTs conducted since the publication of recommendations also supported alternative conclusions. An Iranian RCT with a debatable methodology showed the benefit of ILE administration in tramadol-poisoned patients as an adjunct to standard care to prevent seizures [108]. Finally, in acutely clozapine-poisoned patients, ILE was shown to improve Glasgow coma score, reverse prolonged QTc interval, and shorten hospital stay length [109]. Of note, all RCTs supported ILE safety or reported no adverse effects related to its administration [87,88,106,107,108,109].

Clinical toxicologists are still skeptical, requesting clarifications on how useful ILE may be for poisonings other than LAST [110,111]. An interesting approach based on the Association of Poison Control Centers (AAPCC) National Poison Data System (NPDS) revealed hundreds of cases of cardiotoxicant poisonings reported in 2010–2015 in which ILE was administered, sometimes even prior to the onset of cardiovascular collapse, and death occurred [112]. Despite a suggested transient improvement in a subset of these patients, adverse effects were observed. The number of published ILE failure cases was estimated to outnumber the published ILE success cases. However, even though an elevated mortality rate was expected in such situations, since ILE is dedicated to treat life-threatening toxicity usually refractory to conventional pharmacological therapies, potential publication biases should not alter conclusions about ILE effectiveness. More extensive basic and clinical high-level research is still awaited.

## 5. Next Challenges

### 5.1. Better Scavengers

ILE investigated for biodetoxification has been limited to total parenteral nutrition emulsions, but the range of emulsion design is broad. Absorptive properties could be optimized for drug sequestration to improve the efficacy of emulsion-based detoxification. Reverse engineering of the principles underlying drug delivery will yield efficient and effective scavengers with higher binding capacities and greater specificity [113,114,115]. Nanotechnology-based systems with diverse sizes, shapes, and compositions have been considered for their modular properties in terms of binding affinity, biodistribution profile, and circulation time.

The emulsion surface, the surfactant type and concentration, the fatty acid chain length, and the electrostatic uptake of charged drugs represent parameters that could be modified to optimize binding functionality to redistribute the offending toxicant from its target to the blood or to induce its metabolism into innocuous or less active metabolites.

### 5.2. Non-Triglyceride Formulations

All parenteral nutrition emulsions contain triglyceride oils, but some emulsions using oils not derived from triglycerides have been demonstrated to have greater potential for sequestration and uptake than LCTs. However, non-triglyceride formulations may not also have the cardiotonic effects advocated as important mechanisms of action in recovery from toxicity [116,117].

### 5.3. Liposomes

Liposomes are spherical vesicles of diameters ranging from tens of nanometers to tens of micrometers with an aqueous core surrounded by one or more concentric phospholipid bilayers [118]. These are well-established drug delivery vehicles. As detoxification therapy, liposomes were first investigated in 1973 as carriers for chelating agents in the setting of heavy metal poisoning [119]. Liposomes can be modified in several ways to sequester drugs or toxicants. The hydrophobic phospholipid-made liposome membrane can be modified with surface charges to electrostatically attract drugs. Polymers such as polyethylene glycol can be attached to these phospholipids to improve biocompatibility and increase the circulation time. The hydrophilic interior can be formulated at a modified pH compared to the surrounding medium and carry metabolic enzymes.

Liposomes possessing transmembrane pH gradients have been studied for their capacity to efficiently entrap and transport drugs in vitro [120], ex vivo [121], and in vivo [122,123,124]. Several liposome detoxification therapies have been formulated to contain an aqueous interior at a modified pH at which the target drug is ionized. Indeed, the uncharged forms of many drugs are hydrophobic, and the ionization of such drugs significantly increases their water solubility. The difference in interior pH creates a concentration gradient for the uncharged drug to diffuse across the membrane into the interior in order to be sequestered from the bloodstream. These pH-gradient liposomes make the back-diffusion process across the lipophilic membrane much slower than lipid droplets. More than 80% of the drugs commonly involved in poisoning are amphiphilic, weak bases and, therefore, suitable candidates for this approach. It has also been demonstrated that the drug interacts both with the surface liposome through electrostatic interactions and with the bilayer through hydrophobic interactions [125,126,127]. Recently, as at the beginning of ILE use, Forster et al. evaluated the effects of adding pH-gradient liposomes to peritoneal dialysis fluid. Dialysate concentrations of several drugs including propranolol, amitriptyline, haloperidol, and phenobarbital were enhanced from orally dosed rats [125]. Finally, liposomes have been evaluated as vehicles to contain several other sequestering agents, antidotes, or metabolizing enzymes [128,129,130]. Promising results with translational potential to the bedside are expected in the near future.

Despite limited studies, liposomes appear to be as effective as ILE for detoxification due to the stronger interactions observed with various tested drugs and comparable recovery profiles from toxicity obtained in animals. The design versatility (pH gradients, surface charges, encapsulated antidotes, lipid composition) makes liposomes more attractive for future detoxification therapies [131]. Additional issues remain to be investigated, including the stability, the influence on drug PK [132], and the in vivo physiological effects [133]. However, liposomes may not be able to provide the required additional cardiotonic effects demonstrated by ILE.

### 5.4. Future Considerations for Detoxification Therapies

To reduce drug concentrations below toxic levels in target tissues, important factors still need to be better understood to predict the dynamics of detoxification using colloidal systems. The time scale of sequestration may be significantly shorter than the time required for drug diffusion through tissues or clearance [115,134].

After drug sequestration by a colloidal system, the reduced free drug concentration will allow its redistribution out of some fast-equilibrating tissues such as the heart and lungs to maintain the equilibrium. However, other tissues may continue to slowly release the drug back into the circulation even after the detoxification therapy has been removed from circulation. Measuring each of these parameters and incorporating all the relevant mechanisms and parameters into PK/PD models would be a useful approach to designing overdose therapies [19,47,135].

Future detoxification agents may function as multi-purpose agents, removing and capturing the harmful toxicant to accelerate its metabolism to non-active compounds and delivering antidotes. Combining different antidotal strategies may synergistically antagonize the effect of the offending compound.

Translation to the bedside will also require the implementation of well-designed in vivo protocols. In several studies, scavenging therapies were administered either before or a few minutes after the toxicant, often injected intravenously. These administration regimens are not representative of acute intoxications, which generally occur outside medical facilities, are not treated immediately, and mostly involve oral ingestion. Reproducing realistic poisoning scenarios in upcoming antidotal studies will be very important before moving forward into humans.

Human cases of lipophilic drug poisoning presenting in extremis are infrequent, but RCTs must be undertaken to enable a more informed evaluation of the ILE role in selected poisonings. In order to improve knowledge in this nascent field, guidance with registry data, including both successful cases of ILE use and otherwise, could prove invaluable. One data registry is accessible using the following link http://www.lipidrescue.org/ (accessed on 25 April 2023).

## 6. Conclusions

Present evidence supports the use of ILE only in LAST and lipophilic cardiotoxicant poisonings when there is an immediate threat to life, and other therapies have proven ineffective. ILE as an antidote remains a nascent field, warranting further preclinical investigations, clinical studies, and systematic reporting of use in humans before extending any recommendation. There is an incomplete understanding of ILE efficacy, mechanisms of action, and safety. We need well-designed RCTs in carefully selected groups of patients to help provide the most effective use of ILE.

## Figures and Tables

**Table 1:** Therapies provided in 2,080,917 human exposures based on the 2021 Annual Report of the National Poison Data System from America’s Poison Centers.

Activated charcoal (single or multiple doses)	35,819
Intubation and mechanical ventilation	19,032
Vasopressors	9833
Cardiopulmonary resuscitation	2662
Hemodialysis	2538
Continuous renal replacement therapy	874
High-dose insulin/glucose	661
Lipid emulsion therapy	403
Cardioversion	374
Pacemaker	266
Extracorporeal membrane oxygenation	127

**Table 2:** Summary of the international recommendations regarding intravenous lipid emulsion therapy in cardiotoxicant poisonings (adapted from Gosselin, S. et al. [84]).

Toxicants	Cardiac Arrest	Life-Threatening Toxicity	Non-Life-Threatening Toxicity
Bupivacaine	Strong (1D)	Strong if refractory (1D)	Neutral
Other local anesthetics	Neutral	Low if refractory (2D)	Neutral
Antiarrhythmics class 1	Neutral	Neutral	NR (2D)
Beta-blockers (fat-soluble)	Neutral	Neutral	NR (2D)
Beta-blockers (non-fat-soluble)	Neutral	NR (2D)	NR (2D)
Olanzapine	Neutral	Neutral	NR (2D)
Other antipsychotics	Neutral	NR (2D)	NR (2D)
Diltiazem and verapamil	Neutral	NR (2D)	NR (2D)
Diphenhydramine	Neutral	NR (2D)	NR (2D)
Baclofen	Neutral	Neutral	NR (2D)
Bupropion	Neutral	Low if refractory (2D)	NR (2D)
Cocaine	Neutral	NR (2D)	NR (2D)
Diphenydramine	Neutral	NR (2D)	NR (2D)
Ivermectin	Neutral	Neutral	NR (2D)
Other insecticides	Neutral	NR (2D)	NR (2D)
Malathion	Neutral	NR (2D)	NR (2D)
Other pesticides	Neutral	NR (2D)	NR (2D)
Lamotrigine	Neutral	NR (2D)	NR (2D)
Amitriptyline	Neutral	Low if refractory (2D)	NR(2D)
Other tricyclicantidepressants	Neutral	NR (2D)	NR (2D)
SSRI	Neutral	Neutral	NR (2D)

LA, local anesthetics; SSRI, selective serotonin reuptake inhibitors; NR, not recommended. Strength of recommendation and level of evidence: level 1, strong recommendation (The course of action is considered appropriate by the large majority of experts with no major dissension. The panel is confident that the desirable effects of adherence to the recommendation outweigh the undesirable effects); level 2, weak/conditional recommendation (The course of action is considered appropriate by the majority of experts, but some degree of dissension exists among the panel. The desirable effects of adherence to the recommendation probably outweigh the undesirable effects); grade D, very low level of evidence (Our estimate of the effect is just a guess, and it is very likely that the true effect is substantially different from our estimate of the effect).

**Table 3:** Adverse effects associated with the long-term administration of lipid emulsions.

1. Dyslipidemia (hypertriglyceridemia)
2. Pancreatitis
3. Anaphylaxis
4. Interference with laboratory results
5. Increased shunt fraction and pulmonary artery pressure
6. Acute respiratory distress syndrome
7. Thrombophlebitis with peripheral administration
8. Fat emboli (pulmonary, cerebral, splenic, placental)
9. Infection risk
10. Increased inflammation, hypercoagulability, thrombocytopenia
11. Modulation in cell-mediated immunity
12. Increased oxidative stress

## Data Availability

Not applicable.

## References

[1] Gummin D.D., Mowry J.B., Beuhler M.C., Spyker D.A., Rivers L.J., Feldman R., Brown K., Nathaniel P.T.P., Bronstein A.C., Weber J.A. (2022). 2021 Annual report of the National Poison Data System© (NPDS) from America’s poison centers: 39th annual report. Clin. Toxicol..

[2] Hoegberg L.C., Bania T.C., Lavergne V., Bailey B., Turgeon A.F., Thomas S.H., Morris M., Miller-Nesbitt A., Mégarbane B., Magder S. (2016). Systematic review of the effect of intravenous lipid emulsion therapy for local anesthetic toxicity. Clin. Toxicol..

[3] Cave G., Harvey M. (2009). Intravenous lipid emulsion as antidote beyond local anesthetic toxicity: A systematic review. Acad. Emerg. Med..

[4] Cave G., Harvey M.G. (2014). Should we consider the infusion of lipid emulsion in the resuscitation of poisoned patients?. Crit. Care.

[5] Levine M., Hoffman R.S., Lavergne V., Stork C.M., Graudins A., Chuang R., Stellpflug S.J., Morris M., Miller-Nesbitt A., Gosselin S. (2016). Systematic review of the effect of intravenous lipid emulsion therapy for non-local anesthetics toxicity. Clin. Toxicol..

[6] Weinberg G.L. (2012). Lipid Emulsion Infusion. Anesthesiology.

[7] Eddleston M., Juszczak E., Buckley N.A., Senarathna L., Mohamed F., Dissanayake W., Hittarage A., Azher S., Jeganathan K., Jayamanne S. (2008). Multiple-dose activated charcoal in acute self-poisoning: A randomised controlled trial. Lancet.

[8] Benson B.E., Hoppu K., Troutman W.G., Bedry R., Erdman A., Höjer J., Mégarbane B., Thanacoody R., Caravati E.M., American Academy of Clinical Toxicology (2013). Position paper update: Gastric lavage for gastrointestinal decontamination. Clin. Toxicol..

[9] Clark B.J., Binswanger I.A., Moss M. (2014). The intoxicated ICU patient. Crit. Care Med..

[10] Russel R.L., Westfall B.A. (1962). Alleviation of barbiturate depression. Anesth. Analg..

[11] Shinaberger J.H., Sehar L., Clayton L.E., Barry K.G., Knowlton M., Goldbaum L.R. (1965). Dialysis for intoxication with lipid soluble drugs: Enhancement of glutethimide extraction with lipid dialysate. Trans. Am. Soc. Artif. Intern. Organs.

[12] Ginn H.E., Anderson K.E., Mercier R.K., Stevens T.W., Matter B.J. (1968). Camphor intoxication treated by lipid dialysis. JAMA.

[13] King L.H., Decherd J.F., Newton J.L., Shires D.L., Bradley K.P. (1970). A clinically efficient and economical lipid dialyzer. Use in treatment of glutethimide intoxication. JAMA.

[14] Whang R., Orndorff M., Papper S. (1970). Lipid-electrolyte dialysis--experimental and preliminary clinical observations. Clin. Toxicol..

[15] Clarke L. (1974). Dialysis of drugs in vitro. Br. J. Clin. Pharmacol..

[16] Krieglstein J., Meffert A., Niemeyer D.H. (1974). Influence of emulsified fat on chlorpromazine availability in rabbit blood. Experientia.

[17] Weinberg G.L., VadeBoncouer T., Ramaraju G.A., Garcia-Amaro M.F., Cwik M.J. (1998). Pretreatment or resuscitation with a lipid infusion shifts the dose-response to bupivacaine-induced asystole in rats. Anesthesiology.

[18] Weinberg G., Ripper R., Feinstein D.L., Hoffman W. (2003). Lipid emulsion infusion rescues dogs from bupivacaine-induced cardiac toxicity. Reg. Anesth. Pain Med..

[19] Fettiplace M.R., Lis K., Ripper R., Kowal K., Pichurko A., Vitello D., Rubinstein I., Schwartz D., Akpa B.S., Weinberg G. (2015). Multi-modal contributions to detoxification of acute pharmacotoxicity by a triglyceride micro-emulsion. J. Control. Release.

[20] Baker M.T., Naguib M. (2005). Propofol: The challenges of formulation. Anesthesiology.

[21] Buys M., Scheepers P.A., Levin A.I. (2015). Lipid emulsion therapy: Non-nutritive uses of lipid emulsions in anaesthesia and intensive care. S. Afr. J. Anaesth. Analg..

[22] Waitzberg D.L., Torrinhas R.S., Jacintho T.M. (2006). New parenteral lipid emulsions for clinical use. J. Parenter. Enteral Nutr..

[23] Hippalgaonkar K., Majumdar S., Kansara V. (2010). Injectable lipid emulsions-advancements, opportunities and challenges. Aaps Pharm. Sci. Tech..

[24] Tamilvanan S. (2004). Oil-in-water lipid emulsions: Implications for parenteral and ocular delivering systems. Prog. Lipid Res..

[25] Shim G., Kim M.-G., Kim D., Park J.Y., Oh Y.-K. (2017). Nanoformulation-based sequential combination cancer therapy. Adv. Drug Deliv. Rev..

[26] Leroux J.-C. (2007). Injectable nanocarriers for biodetoxification. Nat. Nanotechnol..

[27] Qu X., Gou M., Zaidan J., Zhang K., Chen S. (2014). Challenges and opportunities in developing nanoparticles for detoxification. Nanomedicine.

[28] Coyle D.E., Denson D.D., Thompson G.A., Myers J.A., Arthur G.R., Bridenbaugh P.O. (1984). The influence of lactic acid on the serum protein binding of bupivacaine: Species differences. Anesthesiology.

[29] Ruan W., French D., Wong A., Drasner K., Wu A.H.B. (2012). A mixed (long- and medium-chain) triglyceride lipid emulsion extracts local anesthetic from human serum in vitro more effectively than a long-chain emulsion. Anesthesiology.

[30] Washington C., Koosha F., Davis S.S. (1993). Physicochemical properties of parenteral fat emulsions containing 20% triglyceride; Intralipid and Ivelip. J. Clin. Pharm. Ther..

[31] Bhal S.K., Kassam K., Peirson I.G., Pearl G.M. (2007). The rule of five revisited: Applying log D in place of log P in drug-likeness filters. Mol. Pharm..

[32] Li Z., Li M., Sun H., Yang Z., Huo Q., Bai Y., Mei Y., Li Y., Quan P., Zhang J. (2022). Prediction of drug capturing by lipid emulsions in vivo for the treatment of a drug overdose. J. Control. Release.

[33] Cave G., Harvey M., Willers J., Uncles D., Meek T., Picard J., Weinberg G. (2014). LIPAEMIC report: Results of clinical use of intravenous lipid emulsion in drug toxicity reported to an online lipid registry. J. Med. Toxicol..

[34] Ozcan M.S., Weinberg G. (2014). Intravenous lipid emulsion for the treatment of drug toxicity. J. Intensive Care Med..

[35] Harvey M., Cave G. (2014). Lipid rescue: Does the sink hold water? And other controversies. Br. J. Anaesth..

[36] Mazoit J.-X., Le Guen R., Beloeil H., Benhamou D. (2009). Binding of long-lasting local anesthetics to lipid emulsions. Anesthesiology.

[37] Laine J., Lokajová J., Parshintsev J., Holopainen J.M., Wiedmer S.K. (2010). Interaction of a commercial lipid dispersion and local anesthetics in human plasma: Implications for drug trapping by “lipid-sinks”. Anal. Bioanal. Chem..

[38] Lokajová J., Holopainen J.M., Wiedmer S.K. (2012). Comparison of lipid sinks in sequestering common intoxicating drugs. J. Sep. Sci..

[39] Hori K., Matsuura T., Mori T., Kuno M., Sawada M., Nishikawa K. (2013). The effect of lipid emulsion on intracellular bupivacaine as a mechanism of lipid resuscitation: An electrophysiological study using voltage-gated proton channels. Anesth. Analg..

[40] Tikhomirov M., Jajor P., Śniegocki T., Poźniak B. (2022). Predicting the efficacy of opioid sequestration by intravenous lipid emulsion using biologically relevant in vitro models of drug distribution. Sci. Rep..

[41] French D., Smollin C., Ruan W., Wong A., Drasner K., Wu A.H.B. (2011). Partition constant and volume of distribution as predictors of clinical efficacy of lipid rescue for toxicological emergencies. Clin. Toxicol..

[42] Weinberg G.L., Ripper R., Murphy P., Edelman L.B., Hoffman W., Strichartz G., Feinstein D.L. (2006). Lipid infusion accelerates removal of bupivacaine and recovery from bupivacaine toxicity in the isolated rat heart. Reg. Anesth. Pain Med..

[43] Weinberg G., Lin B., Zheng S., Di Gregorio G., Hiller D., Ripper R., Edelman L., Kelly K., Feinstein D. (2010). Partitioning effect in lipid resuscitation: Further evidence for the lipid sink. Crit. Care Med..

[44] Niiya T., Litonius E., Petäjä L., Neuvonen P.J., Rosenberg P.H. (2010). Intravenous lipid emulsion sequesters amiodarone in plasma and eliminates its hypotensive action in pigs. Ann. Emerg. Med..

[45] Litonius E., Niiya T., Neuvonen P.J., Rosenberg P.H. (2012). No antidotal effect of intravenous lipid emulsion in experimental amitriptyline intoxication despite significant entrapment of amitriptyline. Basic Clin. Pharmacol. Toxicol..

[46] Harvey M., Cave G., Hoggett K. (2009). Correlation of plasma and peritoneal diasylate clomipramine concentration with hemodynamic recovery after Intralipid infusion in rabbits. Acad. Emerg. Med..

[47] Kuo I., Akpa B.S. (2013). Validity of the lipid sink as a mechanism for the reversal of local anesthetic systemic toxicity. Anesthesiology.

[48] Harvey M., Cave G., Ong B. (2014). Intravenous lipid emulsion-augmented plasma exchange in a rabbit model of clomipramine toxicity; survival, but no sink. Clin. Toxicol..

[49] Harvey M., Cave G. (2012). Case report: Successful lipid resuscitation in multi-drug overdose with predominant tricyclic antidepressant toxidrome. Int. J. Emerg. Med..

[50] French D., Armenian P., Ruan W., Wong A., Drasner K., Olson K.R., Wu A.H. (2011). Serum verapamil concentrations before and after Intralipid^®^ therapy during treatment of an overdose. Clin. Toxicol..

[51] Shi K., Xia Y., Wang Q., Wu Y., Dong X., Chen C., Tang W., Zhang Y., Luo M., Wang X. (2013). The effect of lipid emulsion on pharmacokinetics and tissue distribution of bupivacaine in rats. Anesth. Analg..

[52] Litonius E., Tarkkila P., Neuvonen P.J., Rosenberg P.H. (2012). Effect of intravenous lipid emulsion on bupivacaine plasma concentration in humans. Anaesthesia.

[53] Heinonen J.A., Litonius E., Backman J.T., Neuvonen P.J., Rosenberg P.H. (2013). Intravenous lipid emulsion entraps amitriptyline into plasma and can lower its brain concentration-An experimental intoxication study in pigs. Basic Clin. Pharmacol. Toxicol..

[54] Heinonen J.A., Litonius E., Salmi T., Haasio J., Tarkkila P., Backman J.T., Rosenberg P.H. (2015). Intravenous lipid emulsion given to volunteers does not affect symptoms of lidocaine brain toxicity. Basic Clin. Pharmacol. Toxicol..

[55] Dureau P., Charbit B., Nicolas N., Benhamou D., Mazoit J.-X. (2016). Effect of Intralipid^®^ on the dose of ropivacaine or levobupivacaine tolerated by volunteers: A clinical and pharmacokinetic study. Anesthesiology.

[56] Perichon D., Turfus S., Gerostamoulos D., Graudins A. (2013). An assessment of the in vivo effects of intravenous lipid emulsion on blood drug concentration and haemodynamics following oro-gastric amitriptyline overdose. Clin. Toxicol..

[57] Harvey M., Cave G., Shaw T. (2013). Effect of intravenous lipid emulsion and octreotide on enteric thiopentone absorption; a pilot study. Clin. Toxicol..

[58] Marwick P.C., Levin A.I., Coetzee A.R. (2009). Recurrence of cardiotoxicity after lipid rescue from bupivacaine-induced cardiac arrest. Anesth. Analg..

[59] Fettiplace M.R., Ripper R., Lis K., Lin B., Lang J., Zider B., Wang J., Rubinstein I., Weinberg G. (2013). Rapid cardiotonic effects of lipid emulsion infusion. Crit. Care Med..

[60] Stehr S.N., Ziegeler J.C., Pexa A., Oertel R., Deussen A., Koch T., Hübler M. (2007). The effects of lipid infusion on myocardial function and bioenergetics in l-bupivacaine toxicity in the isolated rat heart. Anesth. Analg..

[61] Gueret G., Pennec J.P., Arvieux C.C. (2007). Hemodynamic effects of intralipid after verapamil intoxication may be due to a direct effect of fatty acids on myocardial calcium channels. Acad. Emerg. Med..

[62] Partownavid P., Umar S., Li J., Rahman S., Eghbali M. (2012). Fatty-acid oxidation and calcium homeostasis are involved in the rescue of bupivacaine-induced cardiotoxicity by lipid emulsion in rats. Crit. Care Med..

[63] Fettiplace M.R., Kowal K., Ripper R., Young A., Lis K., Rubinstein I., Bonini M., Minshall R., Weinberg G. (2016). Insulin signaling in bupivacaine-induced cardiac toxicity: Sensitization during recovery and potentiation by lipid emulsion. Anesthesiology.

[64] Van de Velde M., DeWolff M., Leather H.A., Wouters P.F. (2000). Effects of lipids on the functional and metabolic recovery from global myocardial stunning in isolated rabbit hearts. Cardiovasc. Res..

[65] Shin I.-W., Hah Y.-S., Kim C., Park J., Shin H., Park K.-E., Ok S.H., Lee H.K., Chung Y.K., Shim H.S. (2014). Systemic blockage of nitric oxide synthase by L-NAME increases left ventricular systolic pressure, which is not augmented further by Intralipid^®^. Int. J. Biol. Sci..

[66] Osanai H., Okumura K., Hayakawa M., Harada M., Numaguchi Y., Mokuno S., Murase K., Matsui H., Toki Y., Ito T. (2000). Ascorbic acid improves postischemic vasodilatation impaired by infusion of soybean oil into canine iliac artery. J. Cardiovasc. Pharmacol..

[67] Stojiljkovic M.P., Zhang D., Lopes H.F., Lee C.G., Goodfriend T.L., Egan B.M. (2001). Hemodynamic effects of lipids in humans. Am. J. Physiol. Regul. Integr. Comp. Physiol..

[68] Ok S.-H., Park C.-S., Kim H.J., Lee S.H., Choi B.-H., Eun S.Y., Kim K.N., Yang S.M., Shin I.W., Choi M.J. (2013). Effect of two lipid emulsions on reversing high-dose levobupivacaine-induced reduced vasoconstriction in the rat aortas. Cardiovasc. Toxicol..

[69] Haastrup A.T., Stepniakowski K.T., Goodfriend T.L., Egan B.M. (1998). Intralipid enhances alpha1-adrenergic receptor mediated pressor sensitivity. Hypertension.

[70] Mottram A.R., Valdivia C.R., Makielski J.C. (2011). Fatty acids antagonize bupivacaine-induced I(Na) blockade. Clin. Toxicol..

[71] Nadrowitz F., Stoetzer C., Foadi N., Ahrens J., Wegner F., Lampert A., Koppert W., de la Roche J., Leffler A. (2013). The distinct effects of lipid emulsions used for “lipid resuscitation” on gating and bupivacaine-induced inhibition of the cardiac sodium channel Nav1.5. Anesth. Analg..

[72] Wagner M., Zausig Y.A., Ruf S., Rudakova E., Gruber M., Graf B.M., Volk T. (2014). Lipid rescue reverses the bupivacaine-induced block of the fast Na+ current (INa) in cardiomyocytes of the rat left ventricle. Anesthesiology.

[73] Moussot P.E., Marhar F., Minville V., Vallé B., Dehours E., Bounes V., Ducassé J.L. (2011). Use of intravenous lipid 20% emulsion for the treatment of a voluntary intoxication of flecainide with refractory shock. Clin. Toxicol..

[74] Carreiro S., Blum J., Hack J.B. (2014). Pretreatment with intravenous lipid emulsion reduces mortality from cocaine toxicity in a rat model. Ann. Emerg. Med..

[75] Rahman S., Li J., Bopassa J.C., Umar S., Iorga A., Partownavid P., Eghbali M. (2011). Phosphorylation of GSK-3β mediates intralipid-induced cardioprotection against ischemia/reperfusion injury. Anesthesiology.

[76] Li J., Fettiplace M., Chen S.-J., Steinhorn B., Shao Z., Zhu X., Li C., Harty S., Weinberg G., Vanden Hoek T.L. (2014). Lipid emulsion rapidly restores contractility in stunned mouse cardiomyocytes: A comparison with therapeutic hypothermia. Crit. Care Med..

[77] Li J., Iorga A., Sharma S., Youn J.Y., Partow-Navid R., Umar S., Cai H., Rahman S., Eghbali M. (2012). Intralipid, a clinically safe compound, protects the heart against ischemia-reperfusion injury more efficiently than cyclosporine-A. Anesthesiology.

[78] Li J., Ruffenach G., Kararigas G., Cunningham C.M., Motayagheni N., Barakai N., Umar S., Regitz-Zagrosek V., Eghbali M. (2017). Intralipid protects the heart in late pregnancy against ischemia/reperfusion injury via Caveolin2/STAT3/GSK-3β pathway. J. Mol. Cell Cardiol..

[79] Lou P.-H., Lucchinetti E., Zhang L., Affolter A., Schaub M.C., Gandhi M., Hersberger M., Warren B.E., Lemieux H., Sobhi H.F. (2014). The mechanism of Intralipid^®^-mediated cardioprotection complex IV inhibition by the active metabolite, palmitoylcarnitine, generates reactive oxygen species and activates reperfusion injury salvage kinases. PLoS ONE.

[80] Fettiplace M.R., McCabe D.J. (2017). Lipid emulsion improves survival in animal models of local anesthetic toxicity: A meta-analysis. Clin. Toxicol..

[81] Nafea O.E., Hassan H.A. (2019). Comparative effectiveness of methylene blue versus intravenous lipid emulsion in a rodent model of amlodipine toxicity. Clin. Toxicol..

[82] Tsujikawa S., Matsuura T., Hori K., Mori T., Kuno M., Nishikawa K. (2018). Superior efficacy of lipid emulsion infusion over serum alkalinization in reversing amitriptyline-induced cardiotoxicity in guinea pig. Anesth. Analg..

[83] Fettiplace M.R., Pichurko A.B. (2021). Heterogeneity and bias in animal models of lipid emulsion therapy: A systematic review and meta-analysis. Clin. Toxicol..

[84] Gosselin S., Hoegberg L.C.G., Hoffman R.S., Graudins A., Stork C.M., Thomas S.H., Stellpflug S.J., Hayes B.D., Levine M., Morris M. (2016). Evidence-based recommendations on the use of intravenous lipid emulsion therapy in poisoning. Clin. Toxicol..

[85] Guyatt G.H., Oxman A.D., Vist G.E., Kunz R., Falck-Ytter Y., Alonso-Coello P., Schünemann H.J., GRADE Working Group (2008). GRADE: An emerging consensus on rating quality of evidence and strength of recommendations. BMJ.

[86] Gosselin S., Morris M., Miller-Nesbitt A., Hoffman R.S., Hayes B.D., Turgeon A.F., Gilfix B.M., Grunbaum A.M., Bania T.C., Thomas S.H. (2015). Methodology for AACT evidence-based recommendations on the use of intravenous lipid emulsion therapy in poisoning. Clin. Toxicol..

[87] Taftachi F., Sanaei-Zadeh H., Sepehrian B., Zamani N. (2012). Lipid emulsion improves Glasgow coma scale and decreases blood glucose level in the setting of acute non-local anesthetic drug poisoning--a randomized controlled trial. Eur. Rev. Med. Pharmacol. Sci..

[88] Gil H.-W., Park J.-S., Park S.-H., Hong S.-Y. (2013). Effect of intravenous lipid emulsion in patients with acute glyphosate intoxication. Clin. Toxicol..

[89] Ludot H., Tharin J.-Y., Belouadah M., Mazoit J.-X., Malinovsky J.-M. (2008). Successful resuscitation after ropivacaine and lidocaine-induced ventricular arrhythmia following posterior lumbar plexus block in a child. Anesth. Analg..

[90] Fettiplace M.R., Akpa B.S., Rubinstein I., Weinberg G. (2015). Confusion about infusion: Rational volume limits for intravenous lipid emulsion during treatment of oral overdoses. Ann. Emerg. Med..

[91] Mirtallo J.M., Dasta J.F., Kleinschmidt K.C., Varon J. (2010). State of the art review: Intravenous fat emulsions: Current applications, safety profile, and clinical implications. Ann. Pharmacother..

[92] Grunbaum A.M., Gilfix B.M., Hoffman R.S., Lavergne V., Morris M., Miller-Nesbitt A., Gosselin S. (2016). Review of the effect of intravenous lipid emulsion on laboratory analyses. Clin. Toxicol..

[93] Aditianingsih D., George Y.W.H. (2014). Guiding principles of fluid and volume therapy. Best Pract. Res. Clin. Anaesthesiol..

[94] Hiller D.B., Gregorio G.D., Ripper R., Kelly K., Massad M., Edelman L., Edelman G., Feinstein D.L., Weinberg G.L. (2009). Epinephrine impairs lipid resuscitation from bupivacaine overdose: A threshold effect. Anesthesiology.

[95] Benhamou D., Mazoit J.-X., Zetlaoui P. (2010). Early administration of lipid rescue after initial signs of local anesthetic-induced systemic toxicity. Ann. Fr. Anesth. Reanim..

[96] Hayes B.D., Gosselin S., Calello D.P., Nacca N., Rollins C.J., Abourbih D., Morris M., Nesbitt-Miller A., Morais J.A., Lavergne V. (2016). Systematic review of clinical adverse events reported after acute intravenous lipid emulsion administration. Clin. Toxicol..

[97] Petersen K.M., Jørgensen N.R., Bøgevig S., Petersen T.S., Jensen T.B., Dalhoff K.P., Christensen M.B. (2018). Effects of high-dose, intravenous lipid emulsion on laboratory tests in humans: A randomized, placebo-controlled, double-blind, clinical crossover trial. Clin. Chem. Lab. Med..

[98] Lee H.M.D., Archer J.R.H., Dargan P.I., Wood D.M. (2015). What are the adverse effects associated with the combination use of intravenous lipid emulsion and extracorporeal membrane oxygenation in the poisoned patient?. Clin. Toxicol..

[99] Jeong J. (2008). Continuous renal replacement therapy circuit failure after antidote administration. Clin. Toxicol..

[100] Sin J.H., Tom A., Toyoda A., Roy N., Hayes B.D. (2018). High-dose intravenous lipid emulsion affecting successful initiation of continuous venovenous hemofiltration and extracorporeal membrane oxygenation. Clin. Toxicol..

[101] Paneta M., Waring W.S. (2019). Literature review of the evidence regarding intravenous lipid administration in drug-induced cardiotoxicity. Expert Rev. Clin. Pharmacol..

[102] Forsberg M., Forsberg S., Edman G., Höjer J. (2017). No support for lipid rescue in oral poisoning: A systematic review and analysis of 160 published cases. Hum. Exp. Toxicol..

[103] Lee S.H., Sohn J.T. The underlying mechanism of lipid emulsion resuscitation for drug toxicity: A narrative review. Korean J. Anesthesiol..

[104] García-Ramos S., Fernandez I., Zaballos M. (2021). Lipid emulsions in the treatment of intoxications by local anesthesics and other drugs. Review of mechanisms of action and recommendations for use. Rev. Esp. Anestesiol. Reanim. (Engl. Ed.).

[105] Lee S.H., Kim S., Sohn J.T. (2023). Lipid emulsion treatment for drug toxicity caused by nonlocal anesthetic drugs in pediatric patients: A narrative review. Pediatr. Emerg. Care.

[106] Yu S., Yu S., Zhang L., Gao Y., Walline J., Lu X., Ma Y., Zhu H., Yu X., Li Y. (2019). Efficacy and outcomes of lipid resuscitation on organophosphate poisoning patients: A systematic review and meta-analysis. Am. J. Emerg. Med..

[107] Pannu A.K., Garg S., Bhalla A., Dhibar D.P., Sharma N. (2022). Lipid emulsion for the treatment of acute organophosphate poisoning: An Open-Label randomized trial. Clin. Toxicol..

[108] Kazemifar A.M., Yazdi Z., Bedram A., Mahmoudi J., Ziaee M. (2021). Effects of intravenous lipid emulsion on tramadol-induced seizure; a randomized clinical trial. Arch. Acad. Emerg. Med..

[109] Lgazzar F.M., Elgohary M.S., Basiouny S.M., Lashin H.I. (2021). Intravenous lipid emulsion as an adjuvant therapy of acute clozapine poisoning. Hum. Exp. Toxicol..

[110] Mullins M.E., Seger D.L. (2020). Antidotal use of lipid emulsion-The pendulum swings. Clin. Toxicol..

[111] Hoffman R.S., Gosselin S., Villeneuve E., Hayes B.D., Hoegberg L.C.G., Smolinske S.C. (2020). Comment on antidotal use of lipid emulsion-The pendulum swings. Clin. Toxicol..

[112] Smolinske S., Hoffman R.S., Villeneuve E., Hoegberg L.C.G., Gosselin S. (2019). Utilization of lipid emulsion therapy in fatal overdose cases: An observational study. Clin. Toxicol..

[113] Muhammad F., Nguyen T.D.T., Raza A., Akhtar B., Aryal S. (2017). A review on nanoparticle-based technologies for biodetoxification. Drug Chem. Toxicol..

[114] Forster V., Leroux J.-C. (2015). Nano-antidotes for drug overdose and poisoning. Sci. Transl. Med..

[115] Damitz R., Chauhan A. (2015). Parenteral emulsions and liposomes to treat drug overdose. Adv. Drug Deliv. Rev..

[116] Varshney M., Morey T.E., Shah D.O., Flint J.A., Moudgil B.M., Seubert C.N., Dennis D.M. (2004). Pluronic microemulsions as nanoreservoirs for extraction of bupivacaine from normal saline. J. Am. Chem. Soc..

[117] Damitz R., Chauhan A. (2015). Kinetically stable propofol emulsions with reduced free drug concentration for intravenous delivery. Int. J. Pharm..

[118] Allen T.M., Cullis P.R. (2013). Liposomal drug delivery systems: From concept to clinical applications. Adv. Drug. Deliv. Rev..

[119] Rahman Y.E., Rosenthal M.W., Cerny E.A. (1973). Intracellular plutonium: Removal by liposome-encapsulated chelating agent. Science.

[120] Dhanikula A.B., Lafleur M., Leroux J.-C. (2006). Characterization and in vitro evaluation of spherulites as sequestering vesicles with potential application in drug detoxification. Biochim. Biophys. Acta.

[121] Dhanikula A.B., Lamontagne D., Leroux J.-C. (2007). Rescue of amitriptyline-intoxicated hearts with nanosized vesicles. Cardiovasc. Res..

[122] Bertrand N., Bouvet C., Moreau P., Leroux J.-C. (2010). Transmembrane pH-gradient liposomes to treat cardiovascular drug intoxication. ACS Nano.

[123] Forster V., Luciani P., Leroux J.-C. (2012). Treatment of calcium channel blocker-induced cardiovascular toxicity with drug scavenging liposomes. Biomaterials.

[124] Forster V., Signorell R.D., Roveri M., Leroux J.-C. (2014). Liposome-supported peritoneal dialysis for detoxification of drugs and endogenous metabolites. Sci. Transl. Med..

[125] Fallon M.S., Chauhan A. (2006). Sequestration of amitriptyline by liposomes. J. Colloid Interface Sci..

[126] Howell B., Chauhan A. (2008). Uptake of amitriptyline and nortriptyline with liposomes, proteins, and serum: Implications for drug detoxification. J. Colloid Interface Sci..

[127] Howell B.A., Chauhan A. (2008). Interaction of cationic drugs with liposomes. Langmuir.

[128] Yigit M.V., Mishra A., Tong R., Cheng J., Wong G.C.L., Lu Y. (2009). Inorganic mercury detection and controlled release of chelating agents from ion-responsive liposomes. Chem. Biol..

[129] Levitskaia T.G., Morris J.E., Creim J.A., Woodstock A.D., Luders T., Curry T.L., Thrall K.D. (2010). Aminothiol receptors for decorporation of intravenously administered ^60^Co in the rat. Health Phys..

[130] Szilasi M., Budai M., Budai L., Petrikovics I. (2012). Nanoencapsulated and microencapsulated enzymes in drug antidotal therapy. Toxicol. Ind. Health.

[131] Cave G., Harvey M., Shaw T., Damitz R., Chauhan A. (2013). Comparison of intravenous lipid emulsion, bicarbonate, and tailored liposomes in rabbit clomipramine toxicity. Acad. Emerg. Med..

[132] Kupetz E., Bunjes H. (2014). Lipid nanoparticles: Drug localization is substance-specific and achievable load depends on the size and physical state of the particles. J. Control. Release.

[133] Bertrand N., Leroux J.-C. (2012). The journey of a drug-carrier in the body: An anatomo-physiological perspective. J. Control. Release.

[134] Salmela L., Washington C. (2014). A continuous flow method for estimation of drug release rates from emulsion formulations. Int. J. Pharm..

[135] Fettiplace M.R., Weinberg G. (2015). Past, present, and future of lipid resuscitation therapy. J. Parenter. Enteral Nutr..

